# The Fatty Acid Synthase Inhibitor Platensimycin Improves Insulin Resistance without Inducing Liver Steatosis in Mice and Monkeys

**DOI:** 10.1371/journal.pone.0164133

**Published:** 2016-10-03

**Authors:** Sheo B. Singh, Ling Kang, Andrea R. Nawrocki, Dan Zhou, Margaret Wu, Stephen Previs, Corey Miller, Haiying Liu, Catherine D. G. Hines, Maria Madeira, Jin Cao, Kithsiri Herath, Liangsu Wang, David E. Kelley, Cai Li, Hong-Ping Guan

**Affiliations:** 1 Departments of Discovery Chemistry, Merck Research Laboratories, 2015 Galloping Hill Rd, Kenilworth, NJ, 07033, United States of America; 2 Department of Cardiometabolic Disease, Merck Research Laboratories, 2015 Galloping Hill Rd, Kenilworth, NJ, 07033, United States of America; 3 Department of Pharmacology, Merck Research Laboratories, 2015 Galloping Hill Rd, Kenilworth, NJ, 07033, United States of America; 4 Department of Imaging and Biomarker, Merck Research Laboratories, 2015 Galloping Hill Rd, Kenilworth, NJ, 07033, United States of America; 5 Department of PKPD, Merck Research Laboratories, 2015 Galloping Hill Rd, Kenilworth, NJ, 07033, United States of America; 6 Department of Translational Imaging Biomarkers, Merck Research Laboratories, 770 Sumneytown Pike, West Point, PA, 19486, United States of America; State University of Rio de Janeiro, BRAZIL

## Abstract

**Objectives:**

Platensimycin (PTM) is a natural antibiotic produced by *Streptomyces platensis* that selectively inhibits bacterial and mammalian fatty acid synthase (FAS) without affecting synthesis of other lipids. Recently, we reported that oral administration of PTM in mouse models (*db/db* and *db/+*) with high *de novo* lipogenesis (DNL) tone inhibited DNL and enhanced glucose oxidation, which in turn led to net reduction of liver triglycerides (TG), reduced ambient glucose, and improved insulin sensitivity. The present study was conducted to explore translatability and the therapeutic potential of FAS inhibition for the treatment of diabetes in humans.

**Methods:**

We tested PTM in animal models with different DNL tones, i.e. intrinsic synthesis rates, which vary among species and are regulated by nutritional and disease states, and confirmed glucose-lowering efficacy of PTM in lean NHPs with quantitation of liver lipid by MRS imaging. To understand the direct effect of PTM on liver metabolism, we performed *ex vivo* liver perfusion study to compare FAS inhibitor and carnitine palmitoyltransferase 1 (CPT1) inhibitor.

**Results:**

The efficacy of PTM is generally reproduced in preclinical models with DNL tones comparable to humans, including lean and established diet-induced obese (eDIO) mice as well as non-human primates (NHPs). Similar effects of PTM on DNL reduction were observed in lean and type 2 diabetic rhesus and lean cynomolgus monkeys after acute and chronic treatment of PTM. Mechanistically, PTM lowers plasma glucose in part by enhancing hepatic glucose uptake and glycolysis. Teglicar, a CPT1 inhibitor, has similar effects on glucose uptake and glycolysis. In sharp contrast, Teglicar but not PTM significantly increased hepatic TG production, thus caused liver steatosis in eDIO mice.

**Conclusions:**

These findings demonstrate unique properties of PTM and provide proof-of-concept of FAS inhibition having potential utility for the treatment of diabetes and related metabolic disorders.

## Introduction

Liver lipid homeostasis is balanced by synthesis and removal of TG in hepatocytes. For TG synthesis, fatty acids are provided by three sources: dietary uptake, *de novo* synthesis in the liver, and reverse transport from peripheral tissues [1]. Fatty acid synthase (FAS) is the enzyme that catalyzes the first step of long chain fatty acid(s) synthesis from acetyl-CoA and malonyl-CoA [2, 3]. The primary product of the FAS reaction is palmitate (C16:0), but stearate (C18:0) and shorter fatty acids may also be produced. FAS is a soluble protein that exists as a homodimer of 273 kDa subunits. The FAS enzyme is ubiquitously expressed in human tissues, with the highest level in liver, adipose tissue, and lung [4]. FAS is a target gene of carbohydrate-responsive element-binding protein (ChREBP) and sterol regulatory element-binding transcription factor 1c (SREBP-1c) and expression of FAS is regulated by glucose and insulin [5, 6]. As the major pathway controlling hepatic fatty acid synthesis, FAS expression levels in the liver are correlated with SREBP-1c levels and liver steatosis in rodents and humans [7]. Thus inhibiting FAS had been proposed as a target for diabetes and liver steatosis [7, 8]. While global knockout of FAS is lethal in mice [9], interestingly, liver-specific knockout of FAS (FASKOL) did not show overt phenotype in lean mice fed with chow diet [10]. Surprisingly, FASKOL mice developed fatty liver, hypoglycemia, hypoinsulinemia, and elevated blood ketone bodies when fed with high carbohydrate rich diet for 28 days [10]. The benefit of FAS deficiency on glucose might be due to shift of energy source. Conceptually, a deficiency of FAS will cause accumulation of malonyl-CoA, inhibiting carnitine palmitoyltransferase 1 (CPT1) and the entry of fatty acyl-CoA into mitochondria, thus suppressing β-oxidation. A decrease in β-oxidation should act to shunt energy metabolism from fatty acid oxidation (FAO) to glucose oxidation, and serve to decrease blood glucose or at least increase glucose utilization. Elevation of blood ketones in FASKOL mice seems paradoxical as does the observation that such mice fed with high carbohydrate diet manifest a rise in liver TG [10]. The biochemical adaptations within FASKOL mice are not fully understood, and regardless, these findings raise concern that a pharmacological approach to inhibiting FAS might lead to, or aggravate clinical liver steatosis.

Dietary carbohydrates can be converted to fatty acids via an enzymatic pathway defined as DNL with triglycerides as an end product. Because FAS sits as a key proximal step within the DNL pathway, it is important to consider the relative contribution of DNL to hepatic lipid metabolism. As reported by Trayhurn, P. et al and Donnelly, K. et al [11, 12], the relative capacity and quantitative importance of DNL to the overall triglyceride pool is both species dependent and regulated by carbohydrate content in the diet. Amongst the three sources of fatty acids input to the liver, the contribution of liver DNL varies dramatically in different species and disease states, with for example 25% in lean mice and 60% in *db/db* mice [11]. While in human and NHPs, DNL contributed 7–9% of plasma fatty acids, it is up to 26% in NAFLD patients with hyperglycemia and hyperinsulinemia [12]. In addition to DNL, liver TG content is regulated by energy expenditure and re-uptake of lipids from the periphery. High dietary intake of fructose such as in high fructose corn syrup containing foods and beverages, promotes DNL and has been linked to incidence of non-alcoholic fatty liver disease and associated metabolic disturbances [13]. In mice, DNL is greatly enhanced in genetic models of overfeeding such as the leptin function deficient *ob/ob* or *db/db* mice or by feeding of a high fructose containing diet [14, 15].

We reported previously *in vitro* and *in vivo* efficacies of Platentismycin (PTM) broad-spectrum Gram-positive antibiotic produced by streptomyces platensis [14]. PTM is a potent and highly selective inhibitor of mammalian FAS. It specifically inhibits fatty acid synthesis but not sterol synthesis in rat primary hepatocytes [14, 16]. Inhibition of FAS would induce the accumulation of substrates levels, including malonyl-CoA which should lead to the inhibition of CPT1, a key enzyme regulating the entry of fatty acids into the mitochondria for oxidation. CPT1 inhibitors lower glucose in multiple T2D rodent models, healthy monkeys and T2D patients [17–19]. However, CPT1 inhibitors cause accumulation of triglycerides (TG) in the liver and abnormality of liver enzymes in rodents [17]. We showed that with chronic treatment, PTM reduced ambient glucose in *db/db* mice, decreased liver TG and enhanced insulin sensitivity in *db/+* mice on high-fructose diet [14]. Considering the above mentioned differences in the contribution of DNL to whole body lipogenesis, liver steatosis, and insulin resistance among different models and species [8], it is necessary to test whether PTM has similar efficacy in humans as observed in various preclinical models. More importantly, confirming the effect of PTM on liver TG content in preclinical models is considered a critical safety check before testing the drug in humans. Thus, we expanded the studies of PTM to different preclinical models to confirm the efficacy and de-risk potential adverse effects of FAS inhibition such as liver steatosis in animals with different tones of DNL. This body of work provides proof-of-concept and derisking FAS inhibition for the treatment of metabolic syndrome.

## Materials and Methods

### Experimental animals

Lean C57BL/6 and established diet-induced obese (eDIO) mice were purchased from Taconic Biosciences (Germantown, NY). Lean *db/+* and *db/db* mice were purchased from Jackson Laboratory (Bar Harbor, ME). Animals were maintained in a 12/12 hour light-dark cycle with free access to food and water in an environment with temperature maintained at 22°C. Animals were maintained on a regular rodent chow diet (Teklad, 7012, 5% dietary fat; 3.75 kcal/g) (Madison, WI), or a high fructose diet (Research Diets, D03012908, 60% kcal), or a high-fat diet (Research Diets, D12492, 60% kcal% dietary fat) (New Brunswick, NJ) with free access to water. Diet and dosing information of acute and chronic mouse studies are summarized in Table 1. At the end of studies, carbon dioxide (10–30% / min) inhalation was used to euthanize mice at the end of studies. The method to verify the death of mice includes no movement and heartbeat. All mice procedures and experimental procedures used in this paper were approved and performed in accordance with the guidelines of the Institutional Animal Care and Use Committee (IACUC) of Merck.

**Table 1:** Summary of *in vivo* studies performed in mice.

Mice	Diets	Doses of PTM
***Db/+***	High fructose diet (Research Diets, D03012908)	10, 30, 100 mpk, BID, p.o. 10, 30, 100 mpk in drinking water for 29 days
**C57BL/6**	Chow diet (Teklad 7012)	10, 30, 100 mpk, QD, p.o. for 17 days
	High fat diet (Research Diets, D12492)	50 mpk, BID, p.o. 100 mpk, BID, p.o. for 4 days 40, 125, 400 μg/h, minipump for 10 days
***Db/db***	Chow diet (Teklad 7012)	3, 10, 30 mpk, BID, p.o. for 16 days

### Chronic treatment of mice with Platensimycin (PTM)

Male eDIO mice were fed with high-fat diet at 6 weeks of age and were used in this study at 24 weeks of age. Male *db/+* and *db/db* mice were treated with vehicle or PTM at 8 weeks of age. Lean C57BL/6 mice were treated with vehicle or PTM at 10–12 weeks of age. For minipump study, eDIO mice of around 16 weeks of age were surgically installed with ALZET Osmotic pump (Cat #2002, Cupertino, CA) for continuous delivery of phosphate buffered saline or PTM. Compounds were dissolved in 0.5% methylcellulose and dose by oral gavage (PO) at a volume of 10 ml/kg body weight. For chronic studies, mice were orally dosed with vehicle twice a day (BID), with one dose given in the morning (~9:00 am) and the other in the afternoon (~5:00 pm) for 5 days before start of experiment to be acclimated to oral dosing (n = 3 per group). Animals that lost more than 1 gram of body weight during the conditioning period were excluded in the study. Then mice were divided into treatment groups based on average body weight and daily food intake. Three treatment groups were vehicle (0.5% methylcellulose), CPT1 inhibitor (Teglicar) at 50 mg/kg body weight (mpk), and PTM at 50 mpk. A positive control, the LXR agonist T0901317 (10 mpk, QD) (Tocris, CAS No: 293754-55-9), was included in the study as comparator. Daily food intake and body weight were measured. On day 10, Magnetic Resonance imaging (MRS) was performed on mice from each treatment group to measure hepatic TG content. On day 16, mice were terminated at 4 hours after the morning dose of compounds. Plasma and liver samples were collected to measure liver TG, plasma levels of TG and beta-hydroxybutyrate (β-HBA), and newly made palmitate as a marker for DNL.

### Quantitation of liver lipid content in mice by in vivo MRS

Male eDIO mice at around 16 wks of age were maintained on high fat diet (60% kcal fat, Research Diets, D12492) and treated with vehicle (0.5% methylcellulose), 50 mpk PTM BID, 50 mpk Teglicar (CPT1 inhibitor) BID, or 10 mpk T0901317 (a LXR agonist) QD for 10 days. Liver triglyceride content of eDIO mice was measured non-invasively using localized ^1^H magnetic resonance spectroscopy (MRS). Mice were anesthetized with 2–2.5% isoflurane and placed in a 30 mm MRI volume coil inside a Bruker 9.4T horizontal bore MRI. Axial and sagittal respiratory-gated RARE images covering the abdomen were acquired and used for voxel placement for the MRS acquisitions. A point resolved spectroscopy sequence (PRESS, TR/TE = 7sec/7ms, NSA = 16) was then used to acquire a localized ^1^H NMR spectrum from a 2×2×2 mm^3^ (8 μl) voxel in the liver, being careful to avoid organ boundaries and large blood vessels. The resulting ^1^H NMR spectra were processed using in-house developed Matlab functions to model the lipid (1.3 ppm) and water (4.7 ppm) resonances The areas under the curve of the two signals were integrated and the liver % TG was calculated as lipid / (water + lipid) ×100%. Spectra from two voxels per subject were acquired and the results averaged.

### Measurement of DNL in mice

The pharmacokinetic / pharmacodynamic (PK/PD) relationship of PTM *in vivo* was measured by assessing DNL in male, lean C57BL/6 mice (3 months old) fed either a regular chow diet or a high fructose diet (60% kcal; D03012908, Research diets) for 2 weeks, or eDIO mice maintained on high fat diet (60% kcal, Research Diets, D12492) for 16 wks (n = 7–8 per group). Control mice were fasted overnight. DNL was quantified using a stable isotope tracer method and mass spectrometric analysis as described [20]. PTM was formulated into water and dosed at indicated doses, orally (p.o.) at 5 ml/kg body weight, 30 minutes before dosing of deuterated water (D_2_O) given intraperitoneally (i.p.) at 20 mpk body weight. Mice were euthanized by CO_2_ asphyxiation 2 hours post D_2_O injection. EDTA plasma was collected via cardiac puncture and livers were collected for analysis of PK and DNL.

### Analytical procedures

The ^2^H-labeling of plasma water was determined as described by Shah et al [21]. Briefly, ^2^H that is present in water is exchanged with the hydrogen that is bound to acetone by incubating 5 μl of plasma or known standards in a 2 ml glass screw-top GC vial at room temperature for 4 hours with 2 μl 10 N NaOH (Fisher Scientific) and 5 μl of acetone (Sigma-Aldrich). The instrument is programmed to inject 5 ul of headspace gas from the GC vial in a splitless mode. Samples were analyzed using a 0.8 min isothermal run (Agilent 5973 MS coupled to a 6890 GC oven fitted with an Agilent DB-17 MS column, 15 m × 250 μm × 0.15 μm, the oven was set at 170°C and helium carrier flow was set at 1.0 ml × min^-1^), acetone elutes at ~ 0.4 min, the mass spectrometer was set to perform selected ion monitoring of m/z 58 and 59 (10 ms dwell time per ion) in the electron impact ionization mode.

The ^2^H-labeling of total plasma palmitate was determined as previously described [22]. Samples were processed in 1.5 ml Eppendorf tubes, 50 μl of plasma was mixed with a known amount of heptadecanoic acid (C17:0) internal standard and 250 μl 1N KOH in 80% ethanol. Samples were heated at 65°C for 1 hour, acidified with 50 μl 6N HCl and then extracted in 150 μl chloroform (note that samples were vigorously mixed for 30 sec and centrifuged at 3000 rpm for 5 min). A 100 μl aliquot of the chloroform (lower layer) was collected and evaporated to dryness under N_2_. The dry residue was reacted with TMS-diazomethane to form fatty acid methyl esters. The ^2^H-labeling of methyl palmitate was determined using an Agilent 5973N-MSD equipped with an Agilent 6890 GC system, a DB17-MS capillary column (30 m × 0.25 mm × 0.25 μm); 2 μl of sample was injected in a 20:1 split. The inlet temperature was set at 250°C and the helium gas carrier flow was set at 1 ml / min. The oven temperature was started at 150°C, raised at 20°C / min to 310°C and held for 6 min. The mass spectrometer is operated in the electron impact mode using selective ion monitoring of m/z 270 and 271 at a dwell time of 10 ms per ion, C17:0 is monitored using m/z 284.

### Calculations

The contribution of DNL was determined as previously described [22–24]. Briefly, the water labeling (i.e. precursor) was maintained at a pseudo steady-state during the initial 24 to 48 hours immediately following the administration of each dose of [^2^H] water. The contribution of palmitate synthesis was determined using the equation:

% newly made palmitate = change in palmitate labeling / (water labeling x n) x 100

[23] where “n” represents the number of exchangeable hydrogens (assumed to equal 22). The relative rates of palmitate synthesis were determined by multiplying the % of newly made palmitate (Eq 1) by the relative concentration of total plasma palmitate.

### Perfused Liver NMR Studies

The perfused liver procedure has been published in detail elsewhere [25] and is summarized briefly here. Male C57BL/6 mice (~3 months old) were used for this study during the light cycle (n = 3–5 per group). Mice were anesthetized with Nembutal (100 mg/kg, IP) prior to surgery and carefully monitored to ensure lack of any pain response. After excision of the liver, mice were euthanized by exsanguination. Following a laparotomy, the portal vein was exposed and cannulated, and the liver was excised and perfused with a pre-oxygenated Krebs-Henseleit bicarbonate buffered solution supplemented with 15 mM [1-^13^C] glucose, 1 mM [1-^13^C] palmitate, 2.5% BSA, and either 100 μM PTM, 100 μM L-711 (a CPT1 inhibitor), or DMSO. The liver was then placed into a custom 20 mm NMR tube and the entire assembly was placed inside a 500 MHz wide-bore NMR spectrometer. ^31^P NMR was initially performed to assess liver viability via ATP and Pi levels. This was followed by serial ^13^C NMR acquisitions (30° pulse, D1 = 560 msec, NS = 800 averages, broadband ^1^H decoupling, 11 min. total) during which the following NMR signals were measured: Glucose C1β (96.8 ppm), Glycogen C1 (100.6 ppm), Lactate C3 (21.1 ppm), Acetate C2 (24 ppm), Palmitate C1 (172 ppm), Triglyceride–COOH (182.7 ppm). The integrals of the above signals were calculated for each NMR spectrum and converted to absolute units (μmoles) by comparison with ^13^C NMR spectra of standard solutions of each metabolite acquired under identical conditions. Using the changes in these NMR-determined concentrations over time, the following fluxes were calculated: net glucose uptake, glycogen synthesis, glycolysis (lactate + acetate production), net palmitate uptake, triglyceride (TG) synthesis. In addition, perfusate samples were collected at the end of each study and were biochemically assayed for β-hydroxybutyrate (β-HBA) (Stanbio Laboratory, Boerne, TX), from which the average rate of FAO was calculated, using the (previously verified) assumption that all perfusate β-HBA was derived from the exogenous [1-^13^C] palmitate.

### NHP studies

Monkeys used in this study were socially housed up to 3 animals of the same dose group or companion animal in adjoining stainless steel wire-bottom cages, except when separation is required. The number of animals, animal procedures, and experimental design for this study have been reviewed and approved by the IACUC of Merck. Daily pan/cage sign observation and daily clinical sign observation during each dosing session (including assessment of injection site(s) and eyes) of each monkey were monitored. On day of study termination, pan/cage sign and clinical sign observations were performed once. During the procedure, care was taken to monitor the wellness and minimize the suffering in the monkeys. After procedures, animals were returned back to the colony.

### DNL studies in lean rhesus monkeys after acute and chronic treatments

Lean cynomolgus monkeys (n = 5), rhesus (n = 4), or aged rhesus monkey (n = 6) were sedated with ketamine as the anesthetic during the procedure and administered with ^2^H_2_O mixed with meal by oral gavage (4.2 ml/kg body weight) and vehicle or PTM (60 mpk) in the morning. At 6 hrs post the first dose, animals were sedated again and given the second dose of vehicle or PTM (60 mpk). At 24 hr post the first dose, all animals were sedated under fasting condition with ketamine as the anesthetic during the procedure and blood samples were drawn for DNL determination. The same procedure was used for DNL analysis for processing plasma samples of rodents and NHPs.

### Chronic study in lean aged rhesus monkeys

The lean aged rhesus monkeys (n = 6) were maintained on yogurt and body weight and food intake were monitored weekly. Vehicle and PTM (60 mpk) were administered via subcutaneous injection BID at approximately 7:00 am and 3:00 pm from Day -28 to Day 35. Animals were fasted overnight and blood was drawn weekly prior to the morning dose by using ketamine as the anesthetic during the procedure. Plasma was used for measuring glucose, C-peptide, insulin, active and total GLP-1, free fatty acids, triglycerides, HDL, LDL, β-HBA, GlycoMark and fructosamine on days -29, -22, -15, -8, -1, 7, 14, 21, 28, 35, 50 and 64 (Day 50, 64 for washout period).

For DNL measurement, 3 blood samples were collected on day -9 (baseline) and day 20. Briefly, animals were sedated at 1 hr post administration of vehicle or PTM and the 1^st^ blood sample was drawn for baseline. All animals were then orally gavaged with D_2_O and the meal at a volume of ~5 ml/kg body weight. At 4 hr post-dose, the animals were sedated again for blood collection for the 2^nd^ blood sample for DNL. Afterwards, animals rested with ad lib access to food. At 5:00 pm, all animals were subjected to afternoon vehicle or PTM treatment in a manner similar to the morning routine. Food was removed and animals were subject to overnight fasting. At 24 hr post the 1st dose, all animals were sedated under fasting condition and blood were drawn to determine baseline levels of FPG, insulin, and other relevant metabolic parameters. A meal was orally gavaged via an orogastric tube. At 1 hr post meal, another blood sample was collected for the 3^rd^ sample for DNL determination.

### MRS imaging of liver fat fraction in rhesus monkeys

Rhesus monkeys (approximately 2 to 3 years at the study start) were assigned to one group of 3 females that received 100 mpk BID of the sodium salt form of PTM in deionized water, at a dosing volume of 0.833 mL/kg body weight. Initially (Study Days 1 to 2) the daily subcutaneous injections were in the dorsal interscapular area. In order to minimize local irritation, the injection locations were subsequently rotated among left and right thoracic dorsal and left and right lumbar dorsal sites (beginning on Study Day 3), and extended to the left and right hindlimbs (beginning on Study Day 28). The BID injections were administered approximately 6 hours between doses.

All monkeys underwent general abdominal Magnetic Resonance Image (MRI) exams at pretest and during study week 4. MRI examinations were conducted under general anesthesia through ketamine with propofol induction, followed by isoflurane gas anesthesia. Maintenance fluid requirements were met through continuous intravenous Lactated Ringer's solution administration. Multi-gradient echo images covering the liver were recorded under anesthesia and maintenance intravenous fluid administration for the purpose of liver fat quantification. Images were reconstructed using a LIPO-Quant (Liver Imaging of Phase-related signal Oscillation and Quantification) plug-in written for OsiriX imaging software to provide fat-fraction and R2* maps. The LIPO-Quant image reconstruction separates fat and water signals based on different echo times to quantify the percent fat per pixel with R2* effects demodulated from the signal. Regions of interest (ROIs) were systematically placed in the lobes of the liver on the fat-fraction maps, using the R2* maps to avoid large vessels. Two ROIs each were placed in the left lateral lobe and right lateral lobe, and one ROI each in the left medial and right medial lobes. The average fat-fraction across the liver for each animal at each time point is reported as Means ± SEM.

### FAS enzymatic activity assay, western blotting, and quantitative RT-PCR

FAS enzymatic activity assay was performed as previously reported [14, 16]. Procedures of western blotting and quantitative RT-PCR were similar as the methods described in [26].

### Statistical Analysis

All data are shown as the means ± SEM. Data were plotted in Prism (Graphpad Software, Inc.) and statistical analysis was performed by using One-way ANOVA Multiple Comparisons. Multiplicity adjusted *P* value for each comparison was reported and *P* ≤ 0.05 (95% confidence interval) was deemed as acceptance of significance. Asterisks denote statistical significance of treatment group compared to vehicle group. *P ≤ 0.05, **P ≤ 0.01, ***P ≤ 0.001.

## Results

### Inhibition of PTM on enzymatic activity and expression of FAS

**Fig 1:**
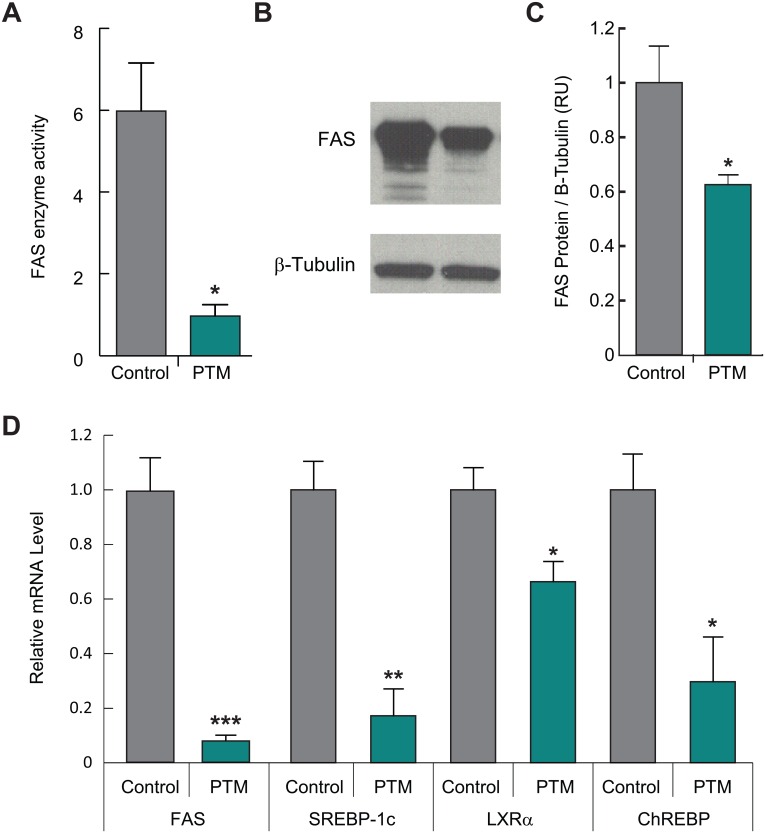
Platensimycin inhibits enzymatic activity and expression of fatty acid synthase (FAS) and lipogenic genes. A: Effect of PTM on FAS enzymatic activity in the liver of eDIO mice treated with PTM (100 mpk, BID, p.o. for 4 days) determined by Malonyl-CoA dependent consumption of NADPH (n = 7–8). B–C: Protein level of FAS in the liver determined by western blot and quantitated by Li-COR. D: Relative RNA levels of FAS, sterol regulatory element-binding transcription factor 1 (SREBP-1c), liver X receptor alpha (LXRα), and carbohydrate-responsive element-binding protein (ChREBP) in the liver determined by quantitative RT-PCR. Bars represent means ± SEM. Asterisks denote statistical significance of treatment group compared to vehicle group. *P ≤ 0.05, **P ≤ 0.01, ***P ≤ 0.001.

PTM is a highly selective inhibitor of FAS in enzymatic activity assay by using biochemically purified proteins and in rat hepatocytes [14, 16]. In this paper, we studied the effect of subchronic treatment of PTM on enzymatic activity and expression of FAS in eDIO mice. With 4-day treatment of PTM (100 mpk, BID, p.o.), FAS protein in the liver was reduced by ~40% (Fig 1A and 1B). This was accompanied by an 83% decrease in FAS enzymatic activity and a 92% decrease in mRNA level (Fig 1C and 1D). In line with this, transcription of other lipogenic genes, SREBP-1c, LXRα, and ChREBP, were also significantly decreased by subchronic treatment of PTM (Fig 1D).

### Comparable inhibition of PTM on DNL in chow diet-fed and high fat diet-fed mice after chronic treatment

**Fig 2:**
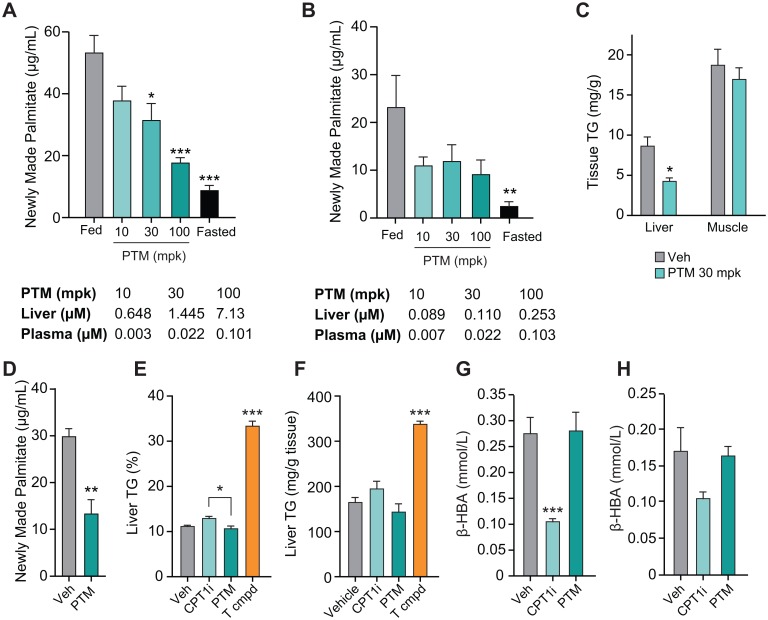
Platensimycin reduces lipogenesis, liver lipid content but has no effect on markers of fatty acid oxidation (FAO) in species with a low de novo lipogenesis (DNL) tone. A–B: Dose-dependent inhibition of PTM on DNL of high fructose-fed *db/+* (A) and chow diet-fed lean C57BL/6 mice (B) (n = 7–8). Concentrations of PTM in liver and plasma are listed under the panels. C: Effect of PTM on tissue TG contents of liver and skeletal muscle of chow diet-fed mice after 17 days of treatment (30 mpk, QD). D–H: Effect of chronic treatment of PTM (50 mpk, BID), CPT1 inhibitor (50 mpk, BID), and T0901317 (10 mpk, QD) on DNL (D), liver TG content, and plasma β-hydroxybutyrate (β-HBA) in diet-induced obese mice. Liver TG contents were analyzed by MRS imaging on day 10 (E) and biochemical methods on day 16 (F). Plasma levels of β-HBA were analyzed at 4 hours post dosing on day 1 after single dose (G) and day 16 after chronic treatment (H) (n = 7). Bars represent means ± SEM. Asterisks denote statistical significance of treatment group compared to vehicle group. *P ≤ 0.05, **P ≤ 0.01, ***P ≤ 0.001.

We have shown previously that liver concentrations of PTM are positively correlated with DNL inhibition in *db/db* mice [14]. Here we show that feeding of C57BL/6 mice with a diet containing 60% fructose increased DNL by 4 to 5 fold relative to fasted states (Fig 2A) as measured by newly synthesized palmitate content. PTM dose dependently reduced DNL with 60% inhibition at a dose of 100 mpk at 2 hours post dosing. Liver concentrations above 1 μM were associated with a significant inhibition of DNL relative to vehicle. PTM accumulates in liver, plasma concentrations are much lower, and accumulation in liver was dependent on the diet and mouse model. As shown in Fig 2B, the overall tone of DNL (ratio between fed and fasted state) was reduced in mice fed a regular chow diet and PTM accumulation in liver was significantly reduced; dosing of 100 mpk did not lead to sufficient liver exposure to effect inhibition of DNL. In conclusion, our data suggest that effectiveness of PTM to inhibit lipid synthesis in the liver is depending on several factors, including the tissue distribution, the disease state, the animal model, and the basal DNL tone. These considerations are important for the estimation of the therapeutic potential of PTM in higher species.

Next we tested the effect of chronic treatment of PTM on tissue lipid content in lean mice. With 17 days of treatment of PTM (30 mpk, p.o., QD), liver TG was decreased by around 50% while no change in skeletal muscle as compared to vehicle treatment (Fig 2C). The effectiveness to reduce liver DNL was then assessed in a chronic study in eDIO mice. Following 16 days of treatment, PTM at 50 mpk administered orally BID significantly inhibited DNL by about 55% (Fig 2D).

**Fig 3:**
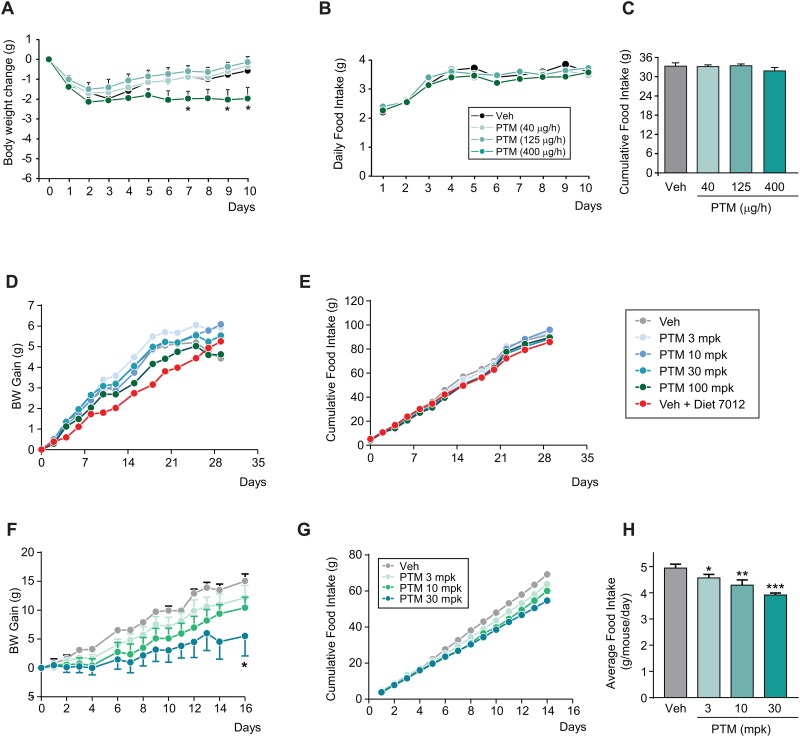
Effect of Platensimycin on body weight gain and food intake in different mouse models. A–C: Subchronic treatment of PTM in eDIO mice (40, 125, and 400 μg/h, minipump for 10 days) (n = 8). D–E: Chronic treatment of PTM in *db/+* mice on high fructose diet (3, 10, 30, and 100 mpk in drinking water for 29 days) (n = 8). F–H: Subchronic treatment of PTM in *db/db* mice for 16 days (3, 10, and 30 mpk, BID, p.o. for 16 days). Bars represent means ± SEM. Asterisks denote statistical significance of treatment group compared to vehicle group. *P ≤ 0.05, **P ≤ 0.01, ***P ≤ 0.001.

As body weight change can significantly alter lipid metabolism, we conducted chronic studies to investigate the effect of PTM treatment on body weight gain and food intake in different mouse models. The body weight of eDIO mice is known to be sensitive to repetitive dosing, thus we used minipump to deliver drug to minimize interference of handling on a daily base. At 400 μg/h, PTM treatment significantly decreased body weight. Interestingly, this reduction in body weight was not accompanied by change in food intake (Fig 3A–3C). The body weight gain of *db/+* mice on high fructose diet showed a trend of reduction in body weight of mice treated with PTM at 100 mpk, while food intake was not affected during the 29-day treatment of PTM in drinking water (Fig 3D and 3E). There was a trend of dose-dependent decrease in body weight gain and food intake in *db/db* mice treated with PTM via oral gavage BID (Fig 3F–3H).

### Parallel comparison of FAS inhibitor and CPT1 inhibitor on liver TG in high fat diet-fed mice after chronic treatment

Blocking of FAO by inhibition of carnitine palmitoyltransferase 1 (CPT1) using Teglicar has been demonstrated to induce hepatic steatosis [17]. An increase of malonyl-CoA also raises a concern of causing fatty liver [17]. To examine this matter, eDIO mice were treated with Teglicar or PTM and tested for hepatic lipid content following chronic treatment. Liver TG was monitored over the course of the study by MRS imaging and by biochemical analysis of the liver tissue at the end of the study. Similar to previous literature [17], Teglicar showed a trend of increased liver TG by day 10. In contrast, this was not observed with PTM. A positive control, the LXR agonist (T0901317), significantly increased liver TG (Fig 2E) [27]. Biochemical analysis of liver tissue for TG content measured at the end of the study revealed similar findings as seen with non-invasive MRS imaging (Fig 2F). To examine the effects of PTM on hepatic FAO *in vivo*, we measured plasma levels of the ketone body β-hydroxybutyrate (β-HBA) as a marker in eDIO mice treated with vehicle or PTM or Teglicar. Teglicar, as expected, significantly decreased plasma β-HBA levels both acutely (2-h post single dose) (Fig 2G) and chronically (Fig 2H). However, PTM at 50 mpk did not change plasma β-HBA, indicating no effect on FAO in eDIO mice.

### Differential effects of CPT1 inhibitor and FAS inhibitor on glucose and lipid metabolism in the liver

**Fig 4:**
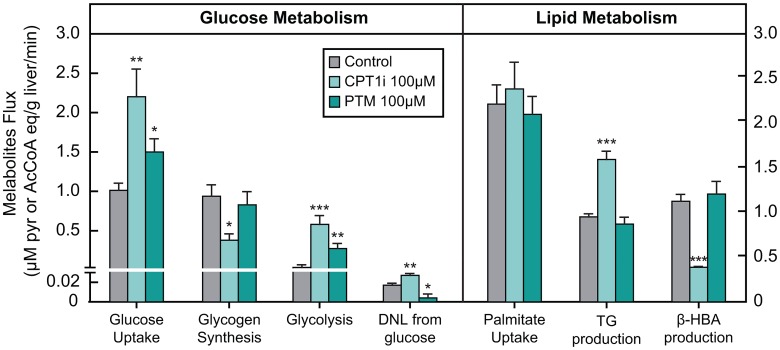
Parallel comparison of PTM and CPT1 inhibitor on glucose and lipid metabolism in perfused liver of lean C57BL/6 mice. PTM and CPT1 inhibitor are used at 100 μM in the perfusion media (n = 3–5 per group). Bars represent means ± SEM. Asterisks denote statistical significance of treatment group compared to control. *P ≤ 0.05, **P ≤ 0.01, ***P ≤ 0.001.

Our *in vivo* studies suggested that FAS inhibition by PTM and a CPT1 inhibitor exert different effects on FAO. To further understand such differences, an *ex vivo* liver perfusion, with tissue isolated from mice chronically treated with vehicle, PTM (50 mpk, BID), or CPT1 inhibitor (50 mpk, BID), was performed. Livers were perfused with vehicle, PTM (100 μM), or CPT1 inhibitor (100 μM). The results of the perfused liver NMR studies are shown in Fig 4. For presentation purposes, all fluxes have been converted to acetyl-CoA or pyruvate units (i.e. 1 glucose = 2 pyruvate, 1 palmitate = 8 AcCoA, etc). Both PTM and CPT1 inhibitor increased hepatic glucose uptake, and this increase was accounted for primarily by increases in glycolysis [14]. Only the CPT1 inhibitor reduced hepatic glycogen synthesis. Neither PTM nor CPT1 inhibitor affected net palmitate uptake. However, the CPT1 inhibitor caused a reduction in hepatic FAO and an increase in hepatic TG synthesis whereas PTM did not cause a re-distribution of palmitate metabolism relative to vehicle. Thus, our findings indicate that PTM potently inhibits DNL and acts to augment hepatic glucose utilization, but does not otherwise influence hepatic palmitate uptake, FAO or rates of esterification into hepatic TG and in these regards differs from the effect of inhibition of CPT1.

### Efficacy of PTM in NHPs

In order to increase confidence in the translation to higher species of the glucose lowering effects seen in rodents, we assessed the effect of PTM in various non-human primate (NHP) models. PTM exhibits suitable PK/PD (DNL) and safety properties to test mechanistic proof of concept in diabetic NHPs. Findings from such studies could indicate the efficacy that might be expected in humans, and provide important insights for clinical study design.

**Fig 5:**
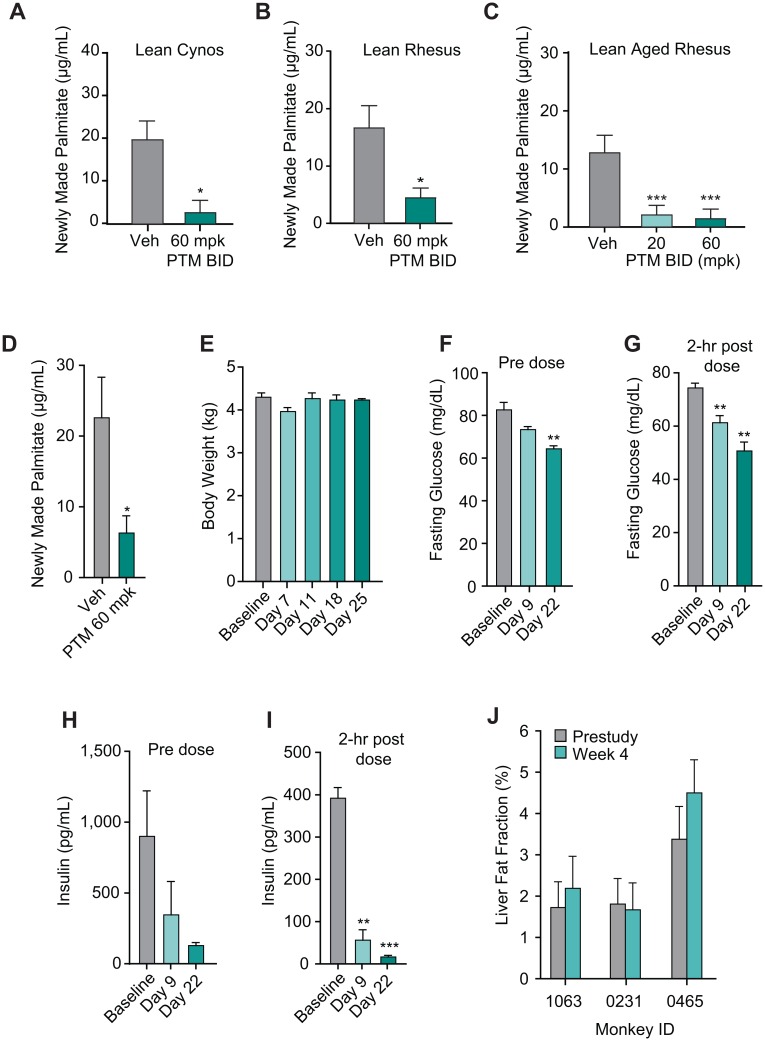
Efficacy of PTM in non-human primates (NHPs). A–C: Inhibition of *de novo* lipogenesis (DNL) by PTM in lean cynomolgus monkeys (A) (n = 5 for vehicle and 4 for PTM) and rhesus (B) (n = 4), and lean aged rhesus monkey (C) (n = 6). Animals were dosed with PTM (60 mpk BID p.o. in A and B, 20 and 60 mpk BID s.c. in C and blood samples were collected at 24 hrs post dosing. D: Effect of PTM on DNL of lean rhesus monkeys (60 mpk, p.o.). E–J: Effect of chronic treatment of PTM on body weight (E), fasting glucose levels for predose (F) and 2 hrs post dose at baseline, day 9 and day 22 (G) and insulin (H–I). PTM was dosed at 60 mpk mixed with yogurt for 28 days. J: Liver fat fraction was determined by MRS imaging at baseline and after 28 days of s.c. dosing of PTM (100 mpk, BID) in lean rhesus monkeys. Bars represent means ± SEM. Asterisks denote statistical significance of treatment group compared to vehicle or baseline. *P ≤ 0.05, **P ≤ 0.01, ***P ≤ 0.001.

The effect of PTM on DNL was first tested in lean cynomolgus and rhesus monkeys at 24 hrs post administration of PTM by oral dosing (60 mpk, p.o., BID). PTM inhibited DNL by 79% in lean cynomolgus monkeys and 73% in lean rhesus monkeys (Fig 5A and 5B). By using a similar protocol, we next tested the ability of PTM to reduce DNL in aged lean rhesus monkeys at 20 and 60 mpk (s.c., BID). A similar reduction of DNL was achieved in rhesus monkeys, with 84% at 20 mpk and 88% at 60 mpk PTM (Fig 5C). After a 28-day treatment with daily dosing of PTM at 60 mpk (PO, BID) in lean rhesus monkeys, PTM suppressed DNL by 70% (Fig 5D), indicating a sustained efficacy in a chronic dosing paradigm. During chronic treatment, there was no significant change in body weight (Fig 5E). Fasting blood glucose levels measured before dosing each day were decreased over time, with glucose on day 22 significantly reduced as compared to each individual animals baseline. Fasting blood glucose levels measured 2 hrs post dose showed similar trend, with glucose on day 9 and 22 significantly reduced as compared to baseline (Fig 5F and 5G). In line with the changes in fasting glucose levels, plasma levels of insulin at pre dose showed a trend of decrease and significantly decreased at 2 hrs post dose on day 9 and 22 (Fig 5H and 5I). Reductions of plasma levels of glucose and insulin indicated that insulin sensitivity of rhesus monkey was improved after chronic treatment with PTM. In a separate study of lean rhesus monkey, liver fat contents from 3 monkeys were quantitated by MRS imaging at baseline and 4 weeks of PTM treatment (100 mpk, s.c., BID). Liver fat fraction was unchanged by chronic treatment of PTM (Fig 5J) relative to lean mass.

## Discussion

Platensimycin is a highly selective inhibitor of mammalian as well as bacterial fatty acid synthesis (FAS). We have recently demonstrated that oral administration of PTM in *db*/*db* and *db*/+ mouse models inhibited DNL, enhanced glucose oxidation, leading to a net reduction of triglyceride and ambient glucose levels accompanied by improved insulin sensitivity. In these mouse models, a large proportion of the hepatic pool of fatty acids derived from DNL [11]. The remaining proportions of hepatic fatty acids are delivered from diet either as a fatty acid ester or free fatty acids. The proportions of fatty acids supplied from various sources are highly variable in different animal models and humans [12]. Moreover, fat contents in diet, especially polysaturated fatty acids such as palmitic acid, play an important role in etiology of liver steatosis via exerting oxidative stress under prolipogenic state [28, 29]. In healthy human as well as non-human primates, the DNL contribution to total lipid is relatively small. However, DNL contribution to total lipids in non-human primates and humans with T2DM and fatty liver disease is significantly higher. Due to the differences in DNL tones of various animal models it is important to know whether findings in an animal model with high DNL tone will translate in an animal model with lower DNL tone and in humans. Therefore, in the current series of experiments, studies of PTM were expanded to include different preclinical models to assess efficacy and evaluate potential adverse effects of FAS inhibition such as liver steatosis.

Teglicar has been reported to increase liver TG without affecting insulin sensitivity [17]. In the head-to-head comparison between PTM and Teglicar, PTM reduced DNL in fructose-diet fed mice a model with low DNL tone in correlation to drug levels in the liver. The effect of PTM on DNL synthesis was less pronounced in chow-fed lean mice though a trend in the reduction of DNL synthesis was consistently observed, a result that was consistent with lower drug levels in liver. Due to selective distribution of PTM into liver and limited distribution of the drug to other tissues (S1 Fig), chow-fed mice only showed significant reduction of liver TG without affecting the TG levels in muscle tissues. In a chronic experiment in eDIO mice to compare PTM and Teglicar side by side, PTM significantly suppressed DNL synthesis whereas Teglicar increased DNL and TG synthesis. Teglicar was also observed to strongly reduce β-oxidation, whereas PTM did not alter rates of β-oxidation by PTM. Taken together these comparative studies point out clear differential effect of FAS inhibition by PTM from CPT1 inhibition by Teglicar and appear to indicate that PTM induced FAS inhibition does not have a strong effect to inhibit CPT1. Further studies will be needed to delineate the molecular mechanisms by which PTM spares inhibition of CPT1 while causing potent inhibition of FAS and the DNL pathway.

The *ex vivo* liver perfusion experiments by NMR indicated that PTM increased glucose uptake. The treatment of perfused livers derived from C57BL/6 mice that were treated chronically with PTM and Teglicar exhibited increased glucose uptake accounted for by an increased rate of glycolysis. This analytical model for the measurement of glycolysis rate may underestimate overall rate of glycolysis since it does not capture conversion of ^13^C-glucose to ^13^C-CO_2_. PTM caused a reduction in DNL from glucose whereas Teglicar exhibited an increase in DNL. This is consistent with the corresponding mechanisms and the *in vivo* observations made with PTM and the CPT1 inhibitor. On the other hand, the increased rate of glycolysis in eDIO model is consistent with the observation made in the high DNL tone *db*/*db* model. Teglicar reduced hepatic glycogen synthesis whereas it was essentially unaffected by PTM. This observation is in sharp contrast with other hepatic glucose uptake mechanisms such as glycogen phosphorylase inhibition [30] and glucokinase overexpression [31], which will directly increase hepatic glycogen levels. Neither PTM nor Teglicar affected net palmitate uptake. However, Teglicar caused a redistribution of palmitate resulting in a reduction in hepatic FAO and increase in hepatic TG synthesis. In contrast PTM had no effect on these fluxes. Palmitate was used as a substrate in the liver perfusion study, so these findings further bolster the interpretation that FAS inhibition, at least as achieved by PTM, does not meaningfully inhibit handling of fatty acids by the liver, including its oxidation, and appears to confine its effects upon DNL. The observed lack of effect of PTM on FAO in eDIO mice in the current studies does seem inconsistent with the data obtained earlier from a *db*/*db* model [14]. Therefore, we postulate that the effect of increased glycolysis may derive, at least in part, from mechanisms other than inhibition of CPT1 caused by increased levels of malonyl-CoA due to FAS inhibition. While these result also differ from FASKOL mice results [10], this difference is not completely surprising since we had not seen liver steatosis in *db/db* and *db/+* models receiving PTM. In fact, we had observed significant reduction of liver TG in *db/db* and *db/+* models [14].

The results obtained from non-human primates which have a low DNL tone confirmed the observations made in rodent models. PTM significantly inhibited DNL in normal lean cynomolgus and rhesus monkeys. The reduction of DNL was even more significant in type 2 diabetic rhesus monkeys, with a maximal effect at a dose of 20 mpk. Chronic treatment of lean aged rhesus monkeys with PTM showed a significant reduction of DNL as well as decreased fasting glucose at peak and trough drug levels, concomitant with decreased plasma insulin levels and without change in body weight and liver fat fraction. These results suggested chronic treatment of PTM improved insulin sensitivity in lean aged NHPs.

In summary, our results demonstrate that antidiabetic effects of our FAS inhibitor, PTM, are not limited to animal models with a comparably high DNL tone. Lipid lowering was observed in models with a low DNL tone including non-human primate, and arguably this is a preclinical model that is more representative of the human physiology. In addition our results in rodents and NHPs suggested that FAS inhibitor does not increase liver TG content, a concern that was raised with CPT1 inhibitors as a potential target for the treatment of diabetes. Our results provide preclinical proof of concept for FAS inhibition as a unique mode of action for the treatment of diabetes. With a highly selective tissue distribution of PTM and concentration in the liver, it is considered a suitable agent for further studies, including clinical studies, to test the hypothesis of an association of diabetes resolution and the treatment of related disorders by FAS inhibition.

## Supporting Information

S1 Fig Distribution of PTM in monkey tissues. PTM was p.o. dosed at 60 mpk at 0 and 2 hrs and monkey was euthanized at 5 hr post the first dosing.
(DOCX)

## Abbreviations

FAS: fatty acid synthase
CPT1: carnitine palmitoyltransferase 1
DNL: *de novo* lipogenesis
PTM: Platensimycin
