# Correction: The Fatty Acid Synthase Inhibitor Platensimycin Improves Insulin Resistance without Inducing Liver Steatosis in Mice and Monkeys

**DOI:** 10.1371/journal.pone.0170721

**Published:** 2017-01-19

**Authors:** Sheo B. Singh, Ling Kang, Andrea R. Nawrocki, Dan Zhou, Margaret Wu, Stephen Previs, Corey Miller, Haiying Liu, Catherine D. G. Hines, Maria Madeira, Jin Cao, Kithsiri Herath, Larry D. Spears, Clay F. Semenkovich, Liangsu Wang, David E. Kelley, Cai Li, Hong-Ping Guan

Dr. Larry D. Spears is not included in the author byline. He should be listed as the thirteenth author and affiliated with the Division of Endocrinology, Metabolism & Lipid Research, Washington University, 660 South Euclid Avenue, St. Louis, MO, 63110, United States of America. The contributions of this author are as follows: methodology and investigation.

Dr. Clay F. Semenkovich is not included in the author byline. He should be listed as the fourteenth author and affiliated with the Division of Endocrinology, Metabolism & Lipid Research, Washington University, 660 South Euclid Avenue, St. Louis, MO, 63110, United States of America. The contributions of this author are as follows: conceptualization and supervision.
