# Supplementary material for: The Fatty Acid Synthase Inhibitor Platensimycin Improves Insulin Resistance without Inducing Liver Steatosis in Mice and Monkeys
Source: PLoS One. 2016 Oct 3;11(10):e0164133. doi: 10.1371/journal.pone.0164133 (PMC5047649; doi:10.1371/journal.pone.0164133)
Supplement: S1 Fig — PTM was p.o. dosed at 60 mpk at 0 and 2 hrs and monkey was euthanized at 5 hr post the first dosing. (DOCX) [file pone.0164133.s001.docx]

**S1 Fig.** Distribution of PTM in monkey tissues. PTM was p.o. dosed at 60 mpk at 0 and 2 hrs and monkey was euthanized at 5 hr post the first dosing.
